# Fault localization in DSLTrans model transformations by combining symbolic execution and spectrum-based analysis

**DOI:** 10.1007/s10270-023-01123-3

**Published:** 2023-09-29

**Authors:** Bentley James Oakes, Javier Troya, Jessie Galasso, Manuel Wimmer

**Affiliations:** 1 DIRO, Université de Montréal, Montréal, Canada; 2 GIGL, Polytechnique Montréal, Montréal, Canada; 3 ITIS Software, Universidad de Málaga, Málaga, Spain; 4 ECE, McGill University, Montréal, Canada; 5 CDL-MINT, Department of Business Informatics - Software Engineering, JKU, Linz, Austria

## Abstract

The verification of model transformations is important for realizing robust model-driven engineering technologies and quality-assured automation. Many approaches for checking properties of model transformations have been proposed. Most of them have focused on the effective and efficient detection of property violations by contract checking. However, there remains the *fault localization* step between identifying a failing contract for a transformation based on verification feedback and precisely identifying the faulty rules. While there exist fault localization approaches in the model transformation verification literature, these require the creation and maintenance of *test cases*, which imposes an additional burden on the developer. In this paper, we combine transformation verification based on *symbolic execution* with spectrum-based fault localization techniques for identifying the faulty rules in DSLTrans model transformations. This fault localization approach operates on the *path condition* output of symbolic transformation checkers instead of requiring a set of test input models. In particular, we introduce a workflow for running the symbolic execution of a model transformation, evaluating the defined contracts for satisfaction, and computing different measures for tracking the faulty rules. We evaluate the effectiveness of spectrum-based analysis techniques for tracking faulty rules and compare our approach to previous works. We evaluate our technique by introducing known mutations into five model transformations. Our results show that the best spectrum-based analysis techniques allow for effective fault localization, showing an average EXAM score below 0.30 (less than 30% of the transformation needs to be inspected). These techniques are also able to locate the faulty rule in the top-three ranked rules in 70% of all cases. The impact of the model transformation, the type of mutation and the type of contract on the results is discussed. Finally, we also investigate the cases where the technique does not work properly, including discussion of a potential pre-check to estimate the prospects of the technique for a certain transformation.

## Introduction

As the adoption of model-driven engineering (MDE) [12] progresses in academia and industry, the validation and verification of *model transformations* is becoming a must in software and systems engineering. These transformations offer a structured approach useful in many model-driven engineering approaches, such as updating models to industry standards [61] or code/documentation generation. However, as these transformations are composed of multiple interacting *rules* which can read and write over the same elements, *model transformation verification* is complex to achieve [67].

*Fault Localization* Many approaches for checking certain properties of model transformations have been proposed in recent years [4, 21, 39, 43, 55]. Most approaches focus on the effective and efficient detection of property violations, such as ensuring that *final states* in a state machine are always transformed into a corresponding *Place* in a Petri Net [43]. However, there is still a research gap on the localization of the model transformation rules to be modified based on the verification feedback. In this context, *fault localization* is to precisely identify the rules which contribute to the failure of a *test case* on that transformation.

For example, consider a transformation rule which should match an input model but instead contains a bug that invalidates a valid match. A *test case* consisting of an *input model* and an *output model* can assist with correctly identifying that the fault is in this rule as opposed to the other rules in the transformation. Fault localization can present a ranked list of the most likely rules to contain a bug, freeing the user from manually inspecting and reasoning about all rules in the transformation. This fault localization problem thus fits within a comprehensive *debugging* process in model transformation verification settings [67]. The research goal is for the transformation engineer to be assisted in quickly locating the faulty rule, as this reduces the overall time needed to fix the bug.

In this paper, we focus on the spectrum-based fault localization (SBFL) technique which has received high interest in program debugging for the localization of bugs [3, 80]. In programming testing, SBFL combines the results of executing test cases and their corresponding code coverage to estimate the likelihood of each program statement of being faulty. In the model transformation context, SBFL examines which rules execute in a given test case, and then assigns “blame” to those rules [69], as further explained in the background section (Sect. 2.4). We term this approach *SBFL-Test* for its reliance on test cases.

While SBFL shows promising results for performing fault localization in model transformations [68], the *SBFL-Test* technique heavily relies on the quality of the test cases available to reach a good and (at the same time) diverse coverage [80]. For example, Troya et al. generated 100 input models for each model transformation [69]. This imposes an additional burden on the user, as these input models must be created and maintained as the transformation evolves [10, 11].

*Symbolic Execution* To eliminate this need for developing and maintaining comprehensive test suites for SBFL [69], we integrate in this paper SBFL with a dedicated model transformation verification approach based on *symbolic execution*. We term this approach *SBFL-Verif*, where the full symbolic state space of a transformation is constructed by reasoning about combinations of rule applications. Then, user-defined *contracts* are proven over the state space to separate the rule combinations which satisfy the contracts from the combinations which do not. This therefore allows the transformation engineer to ensure that the transformation is working as expected without the burden of having to define or generate a single test case.

The particular symbolic execution tool we examine here is SyVOLT [40, 48, 64]. SyVOLT takes as input a model transformation and contracts, and will output whether or not the contracts hold on the transformation. In the context of *fault localization*, SyVOLT does offer some basic debugging information on missing elements and rules present in all failing state spaces to indicate why the contract does not hold [51]. However, this information is not rigorous enough to precisely identify the faulty rules and the transformation engineer must still invest significant time and effort to perform fault localization.

*Contributions and Research Questions* This paper therefore integrates SBFL techniques with the results of symbolic execution verification to create a fault localization approach we term *SBFL-Verif*. The intention is to provide an alternative approach to creating and maintaining test cases for the transformation, and instead use the static approach of symbolically creating a transformation state space. Therefore, the main contributions of this paper are: (a) an adaptation of the SBFL approach to the symbolic execution verification results, and (b) an empirical evaluation of the approach on a set of example model transformations.

We will address the following three knowledge questions, as further detailed in Sect. 4.1:

- **RQ1**—*Is* SBFL-Verif *powerful enough to discover faulty rules effectively?*
- **RQ2**—*How do the different inputs to* SBFL-Verif *(model transformation, contracts and specific faults) affect the results?*
- **RQ3**—*Is* SBFL-Verif *fast enough in practice to discover faulty rules efficiently?*

The remainder of this paper is structured as follows. In Sect. 2, we provide the necessary background material on symbolic execution of model transformations and SBFL in model transformations. The proposed *SBFL-Verif* approach and methodology is then presented in Sect. 3. Section 4 details our research questions and study setup. In particular, we describe the five model transformations used in the evaluation, along with the mutant generation procedure which provides simulated bugs in each transformation. In Sect. 5, we present the results of applying our approach and answer the three research questions. Section 6 discusses our findings, positions our *SBFL-Verif* approach against *SBFL-Test* and examines the threats to validity. Related work is discussed in Sect. 7, while Sect. 8 concludes with an outlook on future work.

## Background

This section introduces the background necessary for this paper. In particular, it briefly presents: (a) the model transformation used as a running example, (b) the DSLTrans transformation language used in our work, (c) the symbolic execution verification approach for proving contracts and the SyVOLT tool which takes as input DSLTrans transformations and (d) the basics of SBFL for model transformations.

### Families2Persons_Extended model transformation

Our running example for explaining the background of our approach is the *Families2Person_Extended* model transformation, which is an extended version of a transformation used for other model transformation verification work [25, 50]. This transformation is composed of 19 rules, and takes *Families* of *Members* who live in *Countries* and *Cities*, and converts those elements into *Men* and *Women* who live in *Communities* and work at *TownHalls*.

**Fig. 1:**
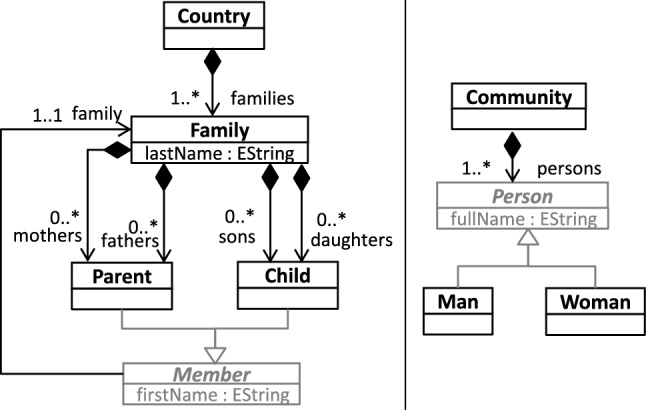
Excerpts of the source and target meta-models of the *Families2Persons_Extended* transformation [50]

An excerpt from the transformation’s input and output *metamodels* (language for the input and output models) is presented in Fig. 1. We refer to [50] for a full description of the transformation.

### DSLTrans transformation language

As the model transformation language used for our verification work, we here present the visual, graph-based DSLTrans [9]. DSLTrans has a number of properties required for our symbolic execution approach such as *termination* (the transformation must finish) and *confluence* (the transformation always produces the same output model for the same input model). As well, DSLTrans is *out-place*, meaning that elements can be created but not rewritten. This ensures that the symbolic state space of a DSLTrans transformation can be made finite. Here, we present a sketch of the relevant semantics of DSLTrans, while the full presentation of the language is provided in [48].

Although the DSLTrans language has restricted expressiveness by design, previous studies have pointed out that the language is applicable to real-world transformation problems which require *translation* or *migration* transformations [58, 59]. These transformations translate (or “evolve”) a model in one meta-model into another meta-model. An example of this is the industrial transformation *GM-to-AUTOSAR* examined in this work (Sect. 4.2.1) where the input model in a proprietary language is translated into a model expressed in an industry standard.

**Fig. 2:**
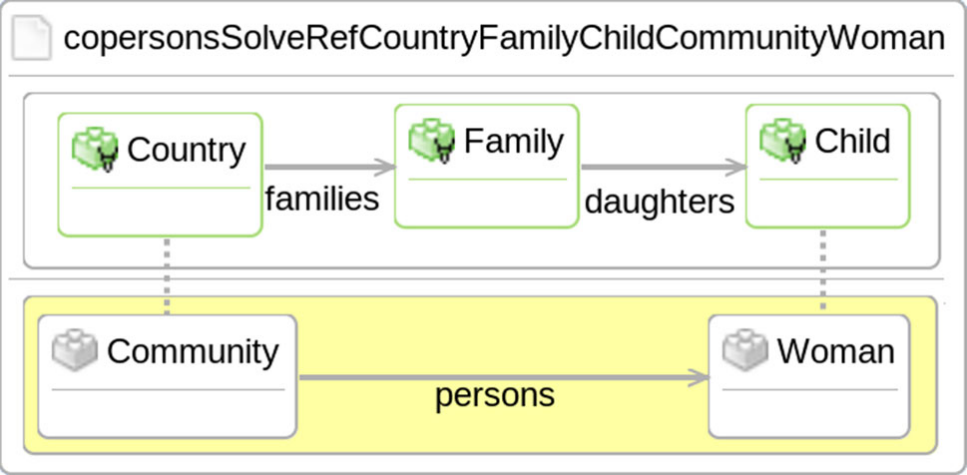
A DSLTrans rule with backward links [48]

DSLTrans transformations operate on *input models* to produce *output models* through the application of *rules* arranged in *layers*. *Rules* are the main building blocks of a DSLTrans transformation. They specify the matching and production of model elements in the input and output models, respectively. The elements inside a rule are grouped by the *MatchModel* and *ApplyModel* components, respectively. In Fig. 2, the *MatchModel* is shown as the white rounded rectangle in the top part, while the *ApplyModel* is depicted in the yellow rounded rectangle in the bottom part of the rule.

When a rule is executed, there is a *match step* followed by *apply step*. In the *match step*, the elements in the *MatchModel* of the rule are matched in the input model along with the connected elements in the *ApplyModel*. In Fig. 2, this would be the *Country*, *Family* and *Child* elements in the *MatchModel* and the *Community* and *Woman* elements in the *ApplyModel*.

If these *match elements* are present, then the *apply step* can begin. In this step, all the elements of the *ApplyModel* which are not linked to the *MatchModel* are instantiated and added to the output model. In Fig. 2, the *persons* association would therefore be created between the matched *Community* and *Woman* elements. Traceability information is also captured by building *traceability links* between newly generated elements and their source elements, i.e., the matched elements of the input model.

DSLTrans provides a *backward link* concept to define rule dependencies by matching over traceability links. For instance, Fig. 2 depicts a rule with two backward links (illustrated by two vertical dotted lines). One backward link is tracing the relationship between the *Country* and *Community* elements, and the other is doing the same for the *Child* and *Woman* elements. These links require that previous rules must have matched on the *Country* and *Child* elements and produced the *Community* and *Woman* elements in a previous layer of the transformation. The rule in Fig. 2 can then correctly create the *persons* association between the previously created elements.

As mentioned, DSLTrans rules are divided into *transformation layers*. DSLTrans can then guarantee confluence through its semantics: (a) all rules of a given layer are fully executed before the next layer is activated, and (b) rules in a given layer cannot match any elements created in that layer [48]. This rigid structure provides the basis for performing layer-by-layer symbolic execution.

### Symbolic execution and contract verification

Here, we describe the symbolic execution and contract verification approach used in this work. As a brief summary, there are two steps to this process. First, *path conditions* (PCs) are created through repeated combination of transformation rules, where the set of PCs for a transformation abstractly represents all possible executions of that transformation.

Second, *structural contracts* (as explained momentarily) given by the user are then proved on those PCs. If a contract fails on a PC, then the rule execution represented by that PC will not satisfy the contract either. Section 3 will detail how the information of which rules were executed in the failing PC is used to localize faults in these rules.

#### Path conditions

*Path conditions* (PCs) represent the symbolic execution of a set of transformation rules. Our symbolic execution approach begins with the initial “empty PC” state which represents all possible executions of the transformation. Then, this state is divided by symbolically applying transformation rules on PCs to create further PCs. These PCs thus contain the input and output elements matched or produced by those rules, and represent a set of transformation executions through an abstraction relation [48]. Note that as DSLTrans is *out-place* and therefore does not permit element modification or deletion, the order of rule application does not need to be considered.

**Fig. 3:**
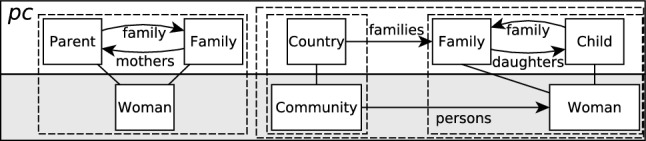
A path condition which represents the application of four transformation rules [48]

For instance, the PC in Fig. 3 represents the symbolic execution of four rules where each rule is denoted by the dashed boxes. Note that one rule is the application of the rule in Fig. 2 which connects the *Community* and *Woman* elements.

The top part of Fig. 3 represents the *input graph* of the PC. That is, the elements which are present in the input model to the transformation if these four rules have executed. The bottom part of Fig. 3 represents the *output graph* of the PC, representing the elements which would be present in the output model. The *traceability links* between the input and output graphs store how the output elements were created from the input elements during the symbolic rule applications.

A PC can therefore be interpreted as: “If only these rules in the transformation were executed, then (as a lower bound) the elements of the input graph of the PC were present in the input model, and the elements of the output graph of the PC are present in the output model.” Though an *abstraction relation*, which abstracts over (a) the number of times a rule has been executed, and (b) the overlap between rule elements, the set of created PCs represent the full state space of the transformation’s execution [48].

#### Contracts and contract verification

The structural contracts used in our symbolic execution technique represent patterns on the input and output models of a model transformation. Just as PCs represent the input and output elements present on a particular branch of a transformation’s execution, so do the contracts check for the presence of these elements. Thus, the structure of contracts is almost identical to that of PCs, and contracts are composed of an *input graph* and an *output graph* with (optional) backward links connecting the two graphs.

**Fig. 4:**
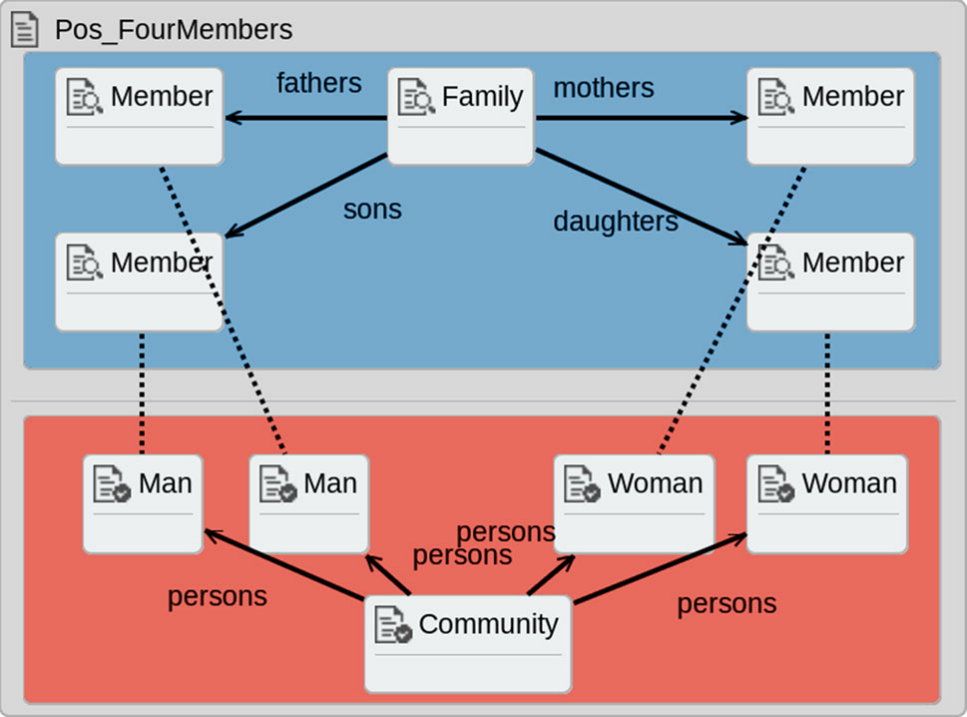
*Pos_FourMembers* contract [48]

Figure 4 shows the *Pos_FourMembers* contract. The *input graph* contains a *Family* connected to four *Members*, while the *output graph* contains a *Community*, two *Men* and two *Women*. Thus, this contract represents the informal statement “A *Family* with a *father*, *mother*, *son* and *daughter* should always produce two *Man* and two *Woman* elements connected to a *Community*”. The backward links (represented by dashed lines) represent the dependency that the output elements must have been produced by a rule that matched over the connected input element.

Contracts are proved on the set of PCs created for a transformation by matching the contract’s pre-condition and post-condition using a graph morphism onto the PCs. For example, the pre-condition of the contract in Fig. 4 is the element in the top half of the contract, while the post-condition is all elements. Our technique determines whether the pre-condition and post-condition hold, which determine whether or not the PC *satisfies* the contract.

That is, contracts are not applicable to all PCs, but only to the ones whose pre-condition matches the elements in the top half of the PCs. If a contract’s pre-condition does not hold on a given PC, then that given PC is termed *not applicable* to that contract [40]. Thus, the contract is neither *satisfied* nor *unsatisfied*. For example, the pre-condition in Fig. 4 contains four *Members*, but the PC in Fig. 3 only contains a *Parent* and a *Child*. This contract therefore *does not apply* to the PC, as it is not appropriate to say the contract is *satisfied* or *unsatisfied*.

If the pre-condition of the contract holds on a PC, but the post-condition does not hold, then the contract is *unsatisfied* and the PC is a counter-example to the contract [40]. This represents that the rule applications covered by the PC do not satisfy the contract. If both the pre-condition and the post-condition hold on a PC, then the contract is *satisfied* by that PC. In this case, whenever the transformation’s input model contains the pattern in the pre-condition, then the pattern contained in the post-condition will hold in the corresponding output model.

These contracts provide assurances for the transformation engineer that patterns in the input model will be correctly transformed into patterns in the output model. If a contract is *satisfied* or *not applicable* for all PCs of the transformation, then the contract holds. Otherwise, the user can be presented with the counter-example PCs where the contract is *unsatisfied*.

The concept of *negative* contracts is also useful to a transformation engineer. These contracts are designed to be *unsatisfied* for a correct transformation. This offers additional expressibility for a user to ensure that the transformation is operating as they expect [51]. In this article, we will not consider negative contracts and all contracts described are expected to hold on a correct transformation.

#### Technique limitations

The symbolic execution nature of our technique implies certain restrictions on contract expressiveness. First, a PC only represents the minimum number of times a rule has been symbolically executed. For example, the PC in Fig. 3 represents that each of those four rules has symbolically executed at least once. This means it is difficult or impossible to represent multiplicity constraints. This is relevant in our *UML-to-ER* example transformation (cf. Section 4.2.1) where the contracts found in [69] deal with individual elements and have been heavily modified to fit the abstractions present in the SyVOLT verification process.

Another restriction imposed by the symbolic execution approach is the lack of validation of input or output attributes. As the contract prover operates on the transformation specification and without a defined input model, PCs can only refer to abstract input attributes. Therefore, a contract for the *Fam-to-Per-Ex* transformation cannot validate that all *Family* elements have a *lastName* that starts with a capital letter.

### SBFL in model transformations

As discussed in Sect. 1, SBFL for model transformations is a technique to identify faulty rules based on failing *test cases* [69]. These test cases are composed of an input model to the transformation and an OCL assertion [73]. As this approach relies on the creation of test input models, let us refer in the remainder of this paper to the approach in [69] as *SBFL-Test*.

This section repeats the details of the *SBFL-Test* approach to constructing *test cases*. The following section (Sect. 3) will then highlight our contribution where the approach is modified to take the results of contract proof on path conditions into account.

#### Building the coverage matrix and error vector

Table 1 depicts an illustrative example showing how *SBFL-Test* is applied for model transformations. The rows of Table 1 represent the rules in the transformation where we are attempting to localize the fault. In this example, we assume that the fault lies in the second rule $tr_2$. The first half of the table’s columns represent ten *test cases*, where each is composed of an input model and the OCL assertion $OCL_2$ [69].

**Table 1:** Example spectrum-based fault localization in the *SBFL-Test* approach. Replicated from [69]

Rule	$tc_{02}$	$tc_{12}$	$tc_{22}$	$tc_{32}$	$tc_{42}$	$tc_{52}$	$tc_{62}$	$tc_{72}$	$tc_{82}$	$tc_{92}$	Susp	Rank
$tr_1$	$\bullet$	$\bullet$	$\bullet$	$\bullet$	$\bullet$	$\bullet$	$\bullet$	$\bullet$	$\bullet$	$\bullet$	0.50	3
$tr_2$ (**BUG**)	$\bullet$		$\bullet$	$\bullet$	$\bullet$	$\bullet$	$\bullet$	$\bullet$	$\bullet$	$\bullet$	1	**1**
$tr_3$	$\bullet$	$\bullet$	$\bullet$	$\bullet$	$\bullet$		$\bullet$		$\bullet$	$\bullet$	0.44	7
$tr_4$	$\bullet$	$\bullet$	$\bullet$	$\bullet$	$\bullet$	$\bullet$	$\bullet$	$\bullet$	$\bullet$	$\bullet$	0.50	3
$tr_5$	$\bullet$	$\bullet$		$\bullet$	$\bullet$	$\bullet$	$\bullet$	$\bullet$	$\bullet$	$\bullet$	0.47	6
$tr_6$	$\bullet$	$\bullet$	$\bullet$	$\bullet$	$\bullet$	$\bullet$	$\bullet$	$\bullet$	$\bullet$	$\bullet$	0.50	3
$tr_7$	$\bullet$						$\bullet$				1	**1**
$tr_8$	$\bullet$	$\bullet$			$\bullet$		$\bullet$	$\bullet$	$\bullet$		0.36	8
$tr_9$		$\bullet$					$\bullet$		$\bullet$		0.18	9
Test Result	F	S	F	F	F	F	F	F	F	F		

If a cell in the table contains $\bullet$ then that test case executes the transformation rule of the particular row. For example, in the test case $tc_{02}$, the transformation rules $tr_1$ to $tr_8$ execute on the input model in that test case. This evaluation results in the *coverage matrix* [1].

The last row of Table 1 represents the *error vector* [1] stating the results of the OCL assertion in a test case on the test model. For *SBFL-Test*, this error vector contains either *successful* (S) or *failed* (F). For example, the *F* result at the bottom of the test case $$tc_{02}$$ means that the OCL assertion $$ OCL _2$$ failed on the input model in $$tc_{02}$$.

This coverage matrix and error vector can then be used to rank the transformation rules in order of *suspiciousness*. This suspiciousness value is always in the range [0, 1] and is calculated from the coverage of the rules by the test cases as explained in Sect. 3. It is assumed that low suspiciousness values indicate a low probability to contain a fault and high values indicate a high probability [69]. The suspiciousness value for each transformation rule in Table 1 is displayed in the “Susp” column which has been computed by the well-known fault localization technique *Tarantula* [34]. The last column then provides a ranking of the rule’s suspiciousness value where top-ranked transformation rules have a higher probability to hold the fault. In this example, the suspiciousness-assigning technique *Tarantula* [34] ranks the faulty transformation rule $tr_2$ as first, in a tie with another rule $tr_7$.

#### EXAM score: effectiveness of suspiciousness techniques

The effectiveness of SBFL approaches, more specifically of suspiciousness techniques, is frequently measured by the EXAM score [81, 82]. The EXAM score in our setting is defined as the percentage of rules in a transformation that has to be investigated until the first faulty rule is found:

$$\begin{aligned} EXAM _{ Score } = \dfrac{ Number~of~rules~examined }{ Total~number~of~rules } \end{aligned}$$

In our illustrative example in Table 1, the faulty rule $tr_2$ is tied in ranking first with the non-faulty rule $tr_7$. Such equally ranked rules may be selected for examination by the user in different orders. For this study, we measure the effectiveness of the techniques in the best-, average- and worst-case scenarios [80], i.e., in the best-case, the faulty rule is selected first while in the worst-case it is selected last. The worst-case EXAM score for this setting is therefore calculated as , which means that  of the transformation rules has to be investigated to find the faulty rule.

The *SBFL-Test* approach has showed good results for fault localization in model transformations [69]. However, the development of appropriate input models for the test cases is challenging [10, 11]. This is the main motivation of the approach we present next, where building these input models is no longer needed. Specifically, we explain how to use our symbolic execution/contract proving technique to build the coverage matrix and error vector for a transformation.

## SBFL-verif approach and methodology

This section presents the *SBFL-Verif* approach for model transformation debugging with the verification feedback obtained from an approach of constructing path conditions and proving structural contracts. As opposed to the *SBFL-Test* approach [69], we do not need *test cases* which include input models for the transformation. Instead, we automatically produce what we call *satisfaction cases*, which are constructed from the path conditions produced in the verification process. For this reason, we refer to the approach of this paper as *SBFL-Verif*, to make a distinction with the *SBFL-Test* approach.

We first detail our contribution of constructing a coverage matrix and the error vector (see Sect. 2.4.1) based on verification feedback from the path condition/contract proving approach. In addition, we describe the adaptation of the suspiciousness calculation procedure for this verification feedback. Subsequently, we present the methodology to apply our approach when debugging a model transformation.

**Table 2:** Tarantula [34] suspiciousness values and ranking when contract $c_3$ fails

Rule	$sc_{03}$	$sc_{13}$	$sc_{23}$	$sc_{33}$	$sc_{43}$	$sc_{53}$	$sc_{63}$	$sc_{73}$	$sc_{83}$	$sc_{93}$	$N_{CF}$	$N_{UF}$	$N_{CS}$	$N_{US}$	$N_{C}$	$N_{U}$	Susp	Rank
$tr_1$	$\bullet$		$\bullet$	$\bullet$	$\bullet$		$\bullet$		$\bullet$		2	1	1	1	6	4	0.57	4
$tr_2$	$\bullet$	$\bullet$	$\bullet$		$\bullet$		$\bullet$			$\bullet$	3	2	2	0	6	4	0.5	5
$tr_3$		$\bullet$			$\bullet$	$\bullet$	$\bullet$	$\bullet$	$\bullet$		3	2	2	0	6	4	0.5	5
$tr_4$ (**BUG**)	$\bullet$		$\bullet$		$\bullet$		$\bullet$	$\bullet$		$\bullet$	4	1	1	1	6	4	0.73	2
$tr_5$		$\bullet$	$\bullet$		$\bullet$			$\bullet$	$\bullet$		4	1	1	1	5	5	0.8	1
$tr_6$	$\bullet$	$\bullet$		$\bullet$				$\bullet$		$\bullet$	2	3	1	1	5	5	0.67	3
Test Result	F	S	F	N	F	N	S	F	F	N								

### Coverage matrix and error vector

This section describes the process for constructing the coverage matrix and error vector required for SBFL from the path conditions (PCs) and contracts in the symbolic execution verification process.

First, the computation of the coverage matrix is based on information about the rules abstracted by every PC, so we take the set of PCs generated by the symbolic execution approach (Sect. 2.3.1): $PC = \{pc_0, pc_1,\ldots , pc_n\}$. Second, the oracle that verifies the PCs is a set of contracts developed by the user (Sect. 2.3.2): $C = \{c_1, c_2,..., c_m\}$. As mentioned in that section, these contracts define the expected elements present in the input models to the transformation and the output models produced by the transformation rule executions.

In our context, we consider a *satisfaction case* as a pair consisting of a PC and a contract: $sc_{ij} = <pc_i, c_j>$. Therefore, our *satisfaction suite* is composed of the Cartesian product of PCs and contracts: $S = PC \times C = \{sc_{01}, sc_{02},..., sc_{nm}\}$. In this setting, the satisfaction case $sc_{ij}$ fails if PC $pc_i$ fails to satisfy contract $c_j$ during the contract proving step.

In the contract proving process, a correct model transformation is denoted by two conditions: a) all contracts must be satisfied by at least one PC, and b) the contract must not be unsatisfied by any PCs. Thus, a contract is not satisfied by a model transformation when there exists at least one PC which fails to satisfy the contract. For instance, for $c_1$ to be satisfied by a model transformation, it has to be satisfied (or not applicable) by $\{sc_{01}, sc_{11},..., sc_{n1}\}$. This is reflected by the error vector. Note that given a PC, not all contracts are satisfied or fail for the PC. In fact, frequently a contract is simply not applicable in a PC, since the contract’s precondition does not match the PC (cf. Section 2.3.2). This is a deviation from the error vector obtained in *SBFL-Test*, where assertions can only *succeed* or *fail*.

Thus from the output of the symbolic execution verification step, we obtain information on which rules are involved in each of the PCs built^1^ and the set of contracts that are satisfied, not satisfied and not applicable by each PC.

In the example shown in Table 2, ten satisfaction cases are aiming to check the correctness of contract $c_3$: $<sc_{03}, sc_{13}, \ldots , sc_{93}>$. We can see in the last row, containing the error vector, that satisfaction case $sc_{03}$ fails (F), which means that contract $c_3$ is not satisfied by PC $pc_0$; satisfaction case $sc_{13}$ is successful (S), meaning that contract $c_3$ is satisfied by PC $pc_1$; and satisfaction case $sc_{33}$ is not applicable (N), which means that the precondition of contract $c_3$ did not match on $pc_3$, and therefore the PC is not applicable.

Note that our notion of *non-applicability* leads to an important divergence regarding which cases are considered satisfied in *SBFL-Test* and *SBFL-Verif*. In *SBFL-Test*, an OCL assertion is checked against instantiated model elements and defines the constraints these elements must satisfy. If an OCL assertion is tested on an input model which does not contain elements targeted by the assertion, then the assertion does not fail and the test case is considered *satisfied*. However, in SBFL-Verif, contracts which do not match the PC precondition are considered not applicable, and thus *neither satisfied nor not satisfied*. To represent this case in the *SBFL-Verif* approach, we explicitly place the *N* marking in the error vectors of the coverage matrix. However, these markings are not taken into account in the computation of the suspiciousness score, as is discussed next.

### Suspiciousness calculation

There exist several techniques which can be applied to rank transformation rules with respect to their suspiciousness scores based on the coverage matrix and error vector. Each technique proposes a different formula for suspiciousness computation (cf. Section 4.2.4). However, all techniques use the same variables in their formula. The values for these variables are computed from the coverage matrix and error vector.

In our case, we group satisfaction cases by contracts, so whenever a contract $c_i$ fails in at least one PC (one in $\{sc_{0i},sc_{1i},...,sc_{ni}\}$), we compute the values of these variables. The notation to refer to the variables is adapted from SBFL in software programs [80] and from *SBFL-Test* [69]:

$$\begin{aligned} \begin{array}{l l} N_{CF} &{} \#\text { failed PCs exercising the rule} \\ N_{UF} &{} \#\text { failed PCs not exercising the rule}\\ N_{CS} &{} \#\text { successful PCs exercising the rule}\\ N_{US} &{} \#\text { successful PCs not exercising the rule}\\ N_{C} &{} \#\text { PCs exercising the rule}\\ N_{U} &{} \#\text { PCs not exercising the rule}\\ N_{S} &{} \#\text { successful PCs}\\ N_{F} &{} \#\text { failed PCs}\\ \end{array} \end{aligned}$$

Table 2 shows the values of $N_{CF}$, $N_{UF}$, $N_{CS}$, $N_{US}$, $N_{C}$ and $N_{U}$ for each transformation rule of our illustrative example. As the values of $N_{S}$ and $N_{F}$ are always the same (two and five, respectively), we do not list them in Table 2. With the help of these values we can compute the suspiciousness of each transformation rule. Let us take the technique used in the table, *Tarantula*, which follows the intuition that statements that are executed primarily by more failed test cases are highly likely to be faulty, while statements that are executed primarily by more successful test cases are less likely to be faulty. The suspiciousness value in this technique is computed as follows: $(N_{CF}/N_F)/(N_{CF}/N_F + N_{CS}/N_S)$). The results are shown in the “Susp” column of the table. As with the *SBFL-Test* approach, these suspiciousness values can then be used to assign a ranking of the most likely rule to contain a fault. As seen in Table 2, the faulty rule has been ranked as second to be examined.

As the suspiciousness techniques have been created for approaches like *SBFL-Test* where assertions can either *succeed* (S) or *fail* (F), they do not incorporate the information that a contract may be *not applicable* (N) in their formulas. Examining the variables above, this would impact $N_{C}$ and $N_{U}$. However, the specific suspiciousness formulas we examine in this work do not take these variables into account (Sect. 4.2.4). Therefore, we leave as a question for future work how to modify these formulas to take into account the *not applicable* designation. At this time, we surmise that this designation does not provide useful information for fault localization, and we continue to use the unmodified suspiciousness calculations found in the *SBFL-Test* approach.

**Fig. 5:**
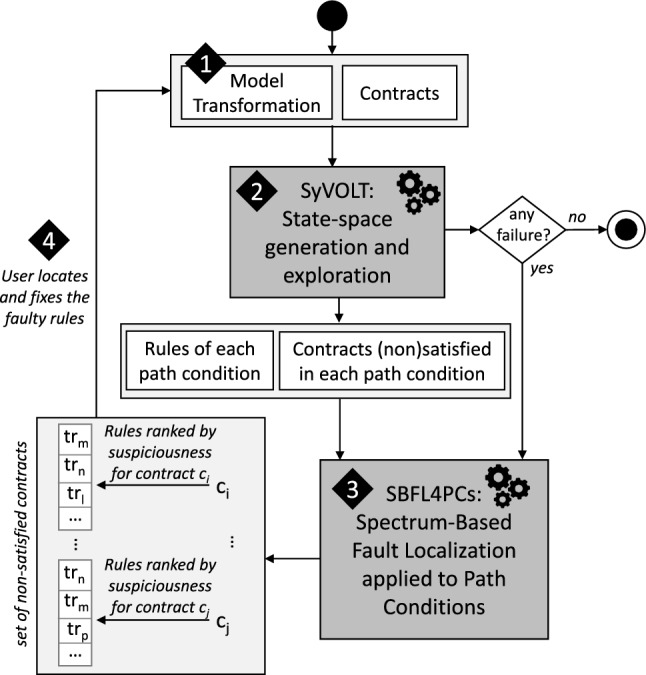
Debugging process of the SBFL-Verif approach (adapted from [69])

### Methodology

The methodology for applying *SBFL-Verif*, adapted from the methodology of *SBFL-Test* [69], is illustrated in Fig. 5 and explained step by step in the following:

1. The *model transformation* and *contracts* (cf. *OCL assertions* in *SBFL-Test*) have to be provided.^2^
2. Our verification tool SyVOLT computes all path conditions comprising the transformation’s state space, determines the rules that are abstracted by each path condition, and checks which contracts are (not) satisfied by each path condition.
3. If there is no failing contract, the process ends. Otherwise, if at least one contract is not satisfied by at least one path condition, the satisfaction cases, coverage matrix, error vector for each non-satisfied contract are constructed from the output of SyVOLT^3^ using our SBFL4PCs tool [63] (cf. Section 4.4). The *suspiciousness-based rankings* of the model transformation rules for each failing contract are returned.
4. The transformation developer investigates the ranking of the rules for one of the failing contracts. Based on the rule ranking and information about the failing contract, the transformation developer aims to improve the transformation by *locating and fixing the bug*.
  Note that SyVOLT also produces debugging output to better identify which elements in the rules and contracts are causing the fault [51]. Thus, our technique localizes faults at the *rule level*, while this (preliminary) debugging information assists in localizing faults *within the rule*.

Once these four steps are performed, the user ends up with a revised model transformation where our assumption is that this version has less bugs than the initial version. Re-iterating the process with the revised model transformation should lead to fewer contracts violation until reaching a final version where all contracts are fulfilled.

#### Methodology comparison to SBFL-Test

In the *SBFL-Test* approach, the input to the fault localization process is the *model transformation*, the test suite of *input models* and the set of *OCL assertions* which provide contracts on the transformation. In the *SBFL-Verif* approach, the path conditions are built directly from the transformation itself. Therefore, there is no need in *SBFL-Verif* to build or maintain a test suite of input models.

Recall from Sect. 2.4 that the *test cases* of the *SBFL-Test* approach were composed of an *input model* and an *OCL assertion*. In the *SBFL-Verif* approach, we instead create *satisfaction cases* composed of a *path condition* and a *contract*.

Once the coverage matrix and error vector are constructed, then the same suspiciousness techniques as in [69] are used to produce the suspiciousness values and rankings.

#### Generalizing SBFL-verif for other transformation languages

The SBFL-Verif approach has currently been implemented only for the DSLTrans language and the SyVOLT contract prover. However, there are no fundamental issues preventing this approach from applying to other model transformation languages. The only requirement is that the language and verification tool produce an analysis similar to path conditions, where (*i*) the path conditions represent the execution of rules in the transformation, and (*ii*) contracts are satisfied or not satisfied on those path conditions.

To apply the SyVOLT tool itself to other model transformation languages, it is currently required to transform the transformation under study into DSLTrans. In the case of ATL, this can be performed using a higher-order transformation (HOT) which we have developed in previous work [49, 50]. This HOT currently is sufficiently reliable to transform many examples from the ATL zoo. The rule application of ATL is made explicit through the layered structure of DSLTrans, and element attributes are represented by the contract equation language present in DSLTrans [48]. For other languages, another HOT would have to be developed from the target language into DSLTrans. Note also that the structure of contracts is not specific to the SyVOLT approach, and similar contracts can be written for other model transformations languages [29, 47].

## Evaluation setup

This section sets up the evaluation of our approach by first defining the research questions (RQs). Then, the experimental setup is explained to detail the different example transformations, contracts, mutants and techniques used for suspiciousness-based rankings computations. The evaluation metrics used for answering each RQ are explained, and the prototype implementation and execution environment are also introduced.

### Research questions

Our main research interest is in studying how the techniques of symbolic execution and SBFL can be combined together as the *SBFL-Verif* approach to effectively identify faulty rules in model transformations. Specifically, we consider that many different variables can influence the quality of the results. Therefore, we aim to answer the following RQs:

***RQ1***—*Is* SBFL-Verif *powerful enough to discover faulty rules effectively?* In Sect. 5.1, we analyze how the *SBFL-Verif* approach performs when being applied on the *satisfaction cases* arising from our symbolic execution/contract proving technique.

In particular, we aim to investigate whether there exist suspiciousness formulas that provide accurate-enough suspiciousness-based rankings.

***RQ2***—*How do the different inputs to* SBFL-Verif *(model transformation, contracts and specific faults) affect the results?* As our approach has different inputs (cf. Section 3.3), we wish to understand how characteristics of these inputs might affect the quality of the results, i.e., the suspiciousness-based rankings. More specifically, this RQ can be divided into the following three sub-questions:

***RQ2.1***—*How do the model transformations under test affect the results quality?* Model transformations are defined in different contexts and can vary in several dimensions such as their size and complexity. Thus, Sect. 5.2.1 addresses how the nature of the model transformation can affect the results.

***RQ2.2***—*How do contracts affect the results quality?* Contracts are the oracle that decide whether a model transformation is correct or not. In this paper, we define two types of contracts in Sect. 4.2.2: *multi-rule* and *single-rule* contracts. An analysis for how the type of contract can affect the results is then presented in Sect. 5.2.2.

***RQ2.3***—*How do specific faults affect the results quality?* Many different types of faults may be present in a model transformation. Thus, we wish to examine how the nature of the faults can affect the results. This is discussed in Sect. 5.2.3.

***RQ3***—*Is the* SBFL-Verif *approach fast enough to discover faulty rules efficiently?* Our model transformation verification tool may construct many path conditions to consider all possibilities (sometimes even thousands of them), which are inputs to our SBFL approach that obtains the suspiciousness-based rankings. We want to study whether the time taken by our approach and accompanying implementation to obtain the rankings is reasonably short enough to be part of a practical model transformation debugging process. This question is answered in Sect. 5.3.

### Experimental setup

Our research questions ask whether we can locate faults in transformations effectively and efficiently. To determine this, it is necessary to have known faults in our transformations. Thus, the rules in our transformations will be mutated simulating a range of faults realistic to those in practice [59]. Each mutant will then be verified for each contract using the SyVOLT contract checker. When a contract is not satisfied, we assume that there is a fault that needs to be localized.

The results of this verification process are presented to the SBFL suspiciousness techniques to identify which rules are the faulty ones. Thus, we will be able to determine if a suspiciousness technique has detected the (known) faulty rule as well as the ranking of the faulty rule in the examination order.

#### Model transformations

For our evaluation of the *SBFL-Verif* approach, we reuse five model transformations from our previous works to evaluate our approach. The first two and last have been sourced from the ATL Zoo repository [8] and converted to their DSLTrans representation [49, 50], while the other two come from previous work in DSLTrans verification [58–60]. An explanation of the domains of each transformation is given in the following.

We acknowledge that a threat to our work is that this selection of model transformations is based on convenience and is not a systematic survey. However, we believe this to be a sufficient selection for two reasons. First, these transformations are comparable in size to those found in related work and a subset of the ATL Zoo repository [46] selected for relevance. Second, while we have developed an automated method for converting ATL transformations into DSLTrans transformations [50], there still remains a significant effort in both developing appropriate contracts for each transformation and (quite ironically) debugging any unintended faults in the transformation.

***RSS-to-ATOM***. This transformation details the production of an ATOM model from an RSS model. ATOM is an XML-based format for synchronizing “feeds” between publishers and consumers; while RSS is a format for syndicating news. The ATL version has been taken from the ATL Zoo [8] and we have obtained the corresponding DSLTrans version using the techniques of [50].

***UML-to-ER***. This transformation is considering structural modeling languages. In particular, entity relationship (ER) diagrams are produced from UML class diagrams. It has been introduced in [76] and extended in [13]. We use the extended version and have applied the technique of [50] to obtain the DSLTrans representation.

***GM-to-AUTOSAR***. This is an industrial transformation used previously in our contract proving work [60]. The transformation receives input models defined in a proprietary legacy meta-model used at General Motors (GM) for vehicle control software development. The output meta-model is the automotive industry standard AUTOSAR^4^. Therefore, this transformation is migrating legacy models to an industrial standard for better tool interoperability [61]. The *GM-to-AUTOSAR* transformation and contracts are further discussed in [58].

***UML-to-Kiltera***. The *UML-to-Kiltera* transformation converts a subset of UML-RT state machines into Kiltera—a language for timed, event-driven, mobile and distributed simulation [58]. The transformation has been proposed in [53] and further developed in [52]. In addition, studying this transformation provided further insights into the contract proving process [59].

***Families-to-Persons_Extended***. The *Families-to-Persons_Extended* transformation (hereafter referred to as *Fam-to-Per-Ex*) has been introduced in Sect. 2.1. As a brief recap, it transforms *Parents* and *Children* in a *Family* into *Men* and *Women* in *Communities*. The original transformation has appeared in previous verification approaches [25] while the *Extended* version has been extensively examined in our previous work [48, 50].

The selected transformations not only differ in their application domains, but also in the complexity of their rules. Table 3 orders the transformations by increasing size and complexity, and reports the number of layers, rules, match elements, apply elements, associations, attributes and backward links. In the three central columns, the number before the slash is in the input component of the rules, while the number after the slash is in the output component. For example, the *Fam-to-Per-Ex* transformation has 46 elements in total in the input components of its rules, and 33 elements in the output components.^5^ The number of rules in the transformations vary between 7 and 19, meaning the largest transformation is almost three times larger than the smallest one.

**Table 3:** Metrics for transformation complexity, rule complexity and number of path conditions produced by the SyVOLT tool

Transformation	Num.	Num.	Num.	Num.	Num.	Num.	Num.
	Layers	Rules	Elements	Assoc.	Attrib.	Backward Links	Path Conds.
*RSS-to-ATOM*	7	7	8/14	1/7	16/14	5	5
*UML-to-ER*	7	9	13/12	4/3	15/8	6	23
*GM-to-AUTOSAR*	8	9	26/20	17/10	5/9	10	5
*UML-to-Kiltera*	8	17	46/91	30/74	25/58	18	558
*Fam-to-Per-Ex*	16	19	46/33	39/14	14/10	24	255

To demonstrate that these transformations are representative, we position their sizes against those in previous SBFL work [69] and in the ATL zoo. The *BibTeX2DocBook* transformation used in previous SBFL work [69] contains nine rules, just as most of our transformations do. Table 4 details statistics of the transformation sizes present in a version of the ATL zoo [46] selected for relevance. The transformations we examine here are therefore as large or larger than a majority of the zoo transformations, with the *UML-to-Kiltera* and *Fam-to-Per-Ex* transformations larger than 70% of the screened zoo.

**Table 4:** Size statistics for relevant transformations in the ATL zoo [46]

Measure	Value
Minimum	5
Mode	5
Quartile_1_	6
Quartile_2_/Median	9
Mean	13.9
Quartile_3_	20
Maximum	31

Table 3 also details the number of path conditions produced by the SyVOLT tool for each transformation. The nonlinear relationship for the size of the transformation and the number of path conditions is due to the nature of the symbolic execution, where rules may overlap or path conditions may be discarded due to violating meta-model constraints (so-called “pruning”) [48]. This nonlinearity means that it is extremely difficult to statically estimate the number of path conditions produced by examining the transformation.

#### Contracts

For each transformation examined in this evaluation, we have collected a set of contracts that must be satisfied for the transformation to be considered correct. As mentioned in Sect. 2.3.2, contracts can be designed to be either satisfied or not satisfied on purpose by a correct transformation. As a reminder, in this evaluation we only consider contracts designed to be satisfied by a correct (non-mutated) version of the transformations.

*Multi- versus Single-Rule Contracts.* As an exploration of how contracts can affect the results of SBFL in the *SBFL-Verif* approach, we offer as a contribution a consideration of two different types of contracts: *multi-rule contracts* and *single-rule contracts*.

The first type is *multi-rule contracts* (MRC), which are sourced from prior work and involve elements from multiple rules in the transformation. For example, the *Pos_FourMembers* contract in Fig. 4 contains elements from four rules involving family members from the *Fam-to-Per-Ex* transformation.

The second type of contracts is termed *single-rule* contracts (SRC). These contracts have been created for this work and are derived directly from the rules of the transformation. That is, the single-rule contracts are produced as one-to-one mirrors of the rules in the transformation, where the intention is to test the production of the elements in that rule.

**Fig. 6:**
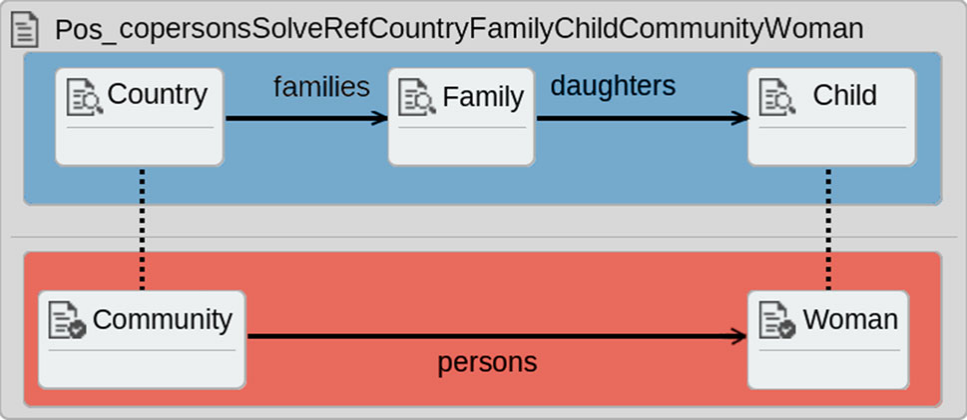
*Single-rule* contract created from the rule in Fig. 2

For example, a single-rule contract for the *Fam-to-Per-Ex* transformation is shown in Fig. 6. This contract is a direct mirror of the elements from the rule in Fig. 2. This contract checks for the proper creation of the *persons* relation in the output model when the appropriate elements are present in the input model.

Single-rule contracts are simple to construct as they are just one-to-one mirrors of transformation rules. The intention for their creation was to explore whether they could be used to immediately identify faulty rules in the fault localization step. However, it was discovered during this work that not all single-rule contracts will hold on the correct version of the transformation. This situation arises because other rules may produce similar input elements such that the contract’s pre-condition will hold, but the post-condition will fail.

As a specific example, one rule (*Hacommittee[...]*) in the *Fam-to-Per-Ex* transformation matches on a *Company* element connected to a *City* element, and produces an *Association* element connected to a *Committee* element. The SRC produced from this rule thus checks for these same two input elements and two output elements. However, another rule (*Htworkers[...]*) also matches on a *Company* element connected to a *City* element (and a *Parent* element), and produces different elements from the *Hacommittee[...]* rule. Thus, whenever the *Htworkers[...]* rule is symbolically executed, and the *Hacommittee[...]* rule is *not* symbolically executed, the SRC produced from the *Hacommittee[...]* rule will not hold.

This example demonstrates that SRCs must be carefully reasoned about to ensure that they are valid contracts for the transformation and that other rules will not interfere. Thus, we conclude that SRCs are easily constructed but are not satisfied in all cases.

**Table 5:** Case study contracts by type (*multi-rule - MRC* or *single-rule - SRC*) and their source

Case study	Contract type	Number	Source
*RSS-to-ATOM*	MRC	0	[48]
	SRC	7	–
*UML-to-ER*	MRC	12	[69]
	SRC	9	–
*GM-to-AUTOSAR*	MRC	7	[58]
	SRC	9	–
*UML-to-Kiltera*	MRC	14	[58]
	SRC	11	–
*Fam-to-Per-Ex*	MRC	6	[50]
	SRC	16	–
Total	MRC	39	
	SRC	52	

Table 5 displays metrics for the 91 contracts considered in this work. For each case study, the number of multi-rule and single-rule contracts is presented, along with the source for the multi-rule contracts^6^. All single-rule contracts were created as part of the current work.

#### Mutant generation

For evaluating our approach, we require a set of transformation rules with known induced faults. To support this, as a contribution of this work we have implemented automatic mutation the SyVOLT verification tool. In particular, it is the rules within the input DSLTrans transformations that are mutated, such as changing the type of an element or adding an association between two elements.

**Table 6:** The mutation operators in our approach, and a classification by type and case study of the mutants which are automatically generated

Mutation Oper.	Description	*RSS-to-ATOM*	*UML-to-ER*	*GM-to AUTOSAR*	*UML-to-Kiltera*	*Families-to-Persons*
Add Association	Adds a valid association between two elements (if not existing)	13	9	27	266	51
Add Backward Link	Adds a backward link (see Sect. 2.2)	0	1	13	102	19
Add Element	Adds a random element from the respective input or output meta-model	161	99	171	1207	437
Delete Element	Deletes an association or element (and connected assocs.)	29	32	82	253	154
Delete Equation	Deletes an equation on elements for matching or setting attribute values	14	17	26	58	10
Modify Equation	Modifies part of an equation, such as removing terms in a concatenation, or replacing involved attributes	0	0	481	42	96
Retype Assoc	Replaces an association’s type by another valid association	3	7	25	436	79
Retype Element	Replaces an element’s type by a type up or down the inheritance hierarchy	5	17	6	277	66
**Total**	**4852**	232	191	840	2658	931

The two left-most columns of Table 6 show the set of implemented mutation operators along with a brief description. These mutation operators were crafted based on the DSLTrans meta-model and experience with common errors made when creating DSLTrans transformation rules [59], similar to the analysis performed in [66].

Note that this set of mutation operators only includes those which impact the symbolic execution technique employed by the SyVOLT contract checker. For example, DSLTrans rules have other constructs such as *Exists* elements and indirect links (described in [48]) which cannot be handled by the current SyVOLT tool. Therefore, these constructs were not considered in our mutation operators. Future work will expand the set of mutation operators to capture other possible transformation errors and constructs.

These mutation operators are applied one-at-a-time to all rules in each transformation, generating hundreds and even thousands of mutants. The total number of mutants generated for each case study, classified by type of mutation, is also displayed in Table 6. In total, 4852 mutant transformations are produced with our approach. Each mutant transformation is then fed as input to the SyVOLT tool, along with the contracts for the case study, following the methodology proposed in Sect. 3.3.

**Table 7:** Average values of mean and standard deviation for EXAM scores in best-, worst- and average-case scenarios

Technique	Mean			Standard deviation		
	BC	WC	AC	BC	WC	AC
Zoltar	.111	.364	**.258**	.186	.238	.199
Kulcynski2	.111	.364	**.258**	.186	.238	.199
Ochiai	.111	.365	**.258**	.186	.238	.198
Op2	.111	.364	**.261**	.209	.238	.209
Russel Rao	.111	.386	**.265**	.187	.228	.194
Mountford	.111	.381	**.270**	.190	.258	.209
Br.-Banq	.111	.386	**.274**	.188	.240	.199
B.-U & B	.111	.408	**.300**	.213	.250	.218
S. Match	.111	.445	.336	.254	.267	.246
Rog & Tan	.111	.445	.336	.254	.267	.246
Tarantula	.111	.511	.338	.190	.322	.215
DStar	.111	.491	.382	.289	.326	.296
Ar. Mean	.111	.842	.503	.204	.317	.196
Phi	.111	.842	.503	.204	.317	.196
Cohen	.111	.842	.503	.204	.317	.196
Ochiai2	.111	.888	.529	.169	.297	.183
Pierce	.111	.951	.662	.317	.192	.201
Barinel	.778	.842	.669	.322	.307	.274

Bold values indicate the scores are acceptable results for an EXAM score

#### Techniques for suspiciousness computation

For a list of techniques, we mirror the extensive catalogue of 18 techniques collected in [69]: *Arithmetic Mean* [81], *Barinel* [2], *Baroni-Urbani & Buser* [79], *Braun-Banquet* [80], *Cohen* [45], D* [78], *Kulczynski2* [45], *Mountford* [79], *Ochiai* [3], *Ochiai2* [7], *Op2* [45], *Phi* [42], *Pierce* [80], *Rogers & Tanimoto* [41], *Russel Rao* [54], *Simple Matching* [80], *Tarantula* [34] and *Zoltar* [32].

For brevity, we refer the reader to our SBFL4PC tool repository [63] for the formulas and descriptions of each technique^7^.

### Evaluation metrics

To answer our research questions, we use several metrics depending on the nature of the RQ.

#### Evaluation metrics for answering RQ1

For answering RQ1 in Sect. 5.1, EXAM scores are employed. As explained in Sect. 2.4.2, there can be ties in the suspiciousness-based rankings [80, 81]. This is the reason why EXAM scores for the best-, worst- and average-case scenarios need to be obtained. For example, Table 7 displays the average values of mean and standard deviation for EXAM scores in best-, worst- and average-case scenarios for each suspiciousness technique.

Lower EXAM scores are preferable as these indicate that the faulty rule will be presented earlier for examination. Consider that the average number of transformation rules in our evaluation is 12.2 (cf. Table 3), and the average number of rules in the ATL zoo is 11.2 (cf. Table 4). In this work, we consider an EXAM score below 0.3 acceptable, which implies that on average the user will be directed toward the top four possible rules to find the bug.

To complement the information provided by the EXAM score, we also want to know the percentage of cases in which the faulty rule is positioned in the top-three rules in the suspiciousness-based rankings (shown in Table 8). We consider that an effective technique locates the faulty rule among the top-three rules it provides, in at least 70% of the cases.

#### Evaluation metrics for answering RQ2

This research question is divided in three sub-questions and is answered in Sect. 5.2. To respond to RQ2.1 about the effects of the transformation, we have obtained the EXAM score values in the average-case scenario, for each suspiciousness technique, for each model transformation under test and both types of contract. These values are presented in Table 9. Techniques are sorted with increasing value of EXAM score, and values below 0.3 have been highlighted.

**Fig. 7:**
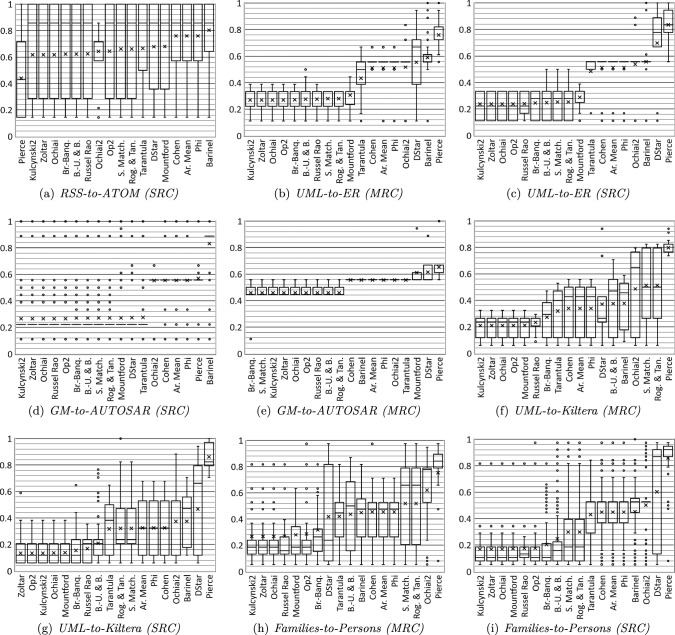
Box-plots of the EXAM scores of each technique per transformation in the average case (MRC: multi-rule contracts; SRC: single-rule contracts)

We have also obtained boxplots with the EXAM score values in the average-case scenario for each model transformation, which are presented in Fig. 7. A boxplot is a standardized way of displaying the distribution of data based on a five-number summary. Techniques in each boxplot have been sorted according to the average value of the EXAM score. Since these boxplots have been grouped by type of contract (multi-rule contracts versus single-rule contracts), they are also used for answering RQ2.2 about how the type of contract affects the results.

For answering RQ2.3 about the effects of the type of mutant on the results, we display in Table 10 the EXAM scores in the average-case scenario for each technique, grouped by the type of mutant.

**Table 8:** Percentage of cases in which the faulty rule is placed within the top-three in the AC scenario

Technique	Perc. Top-3 (%)	Technique	Perc. Top-3 (%)
Kulcynski2	**74.56**	Rog & Tan	65.44
Zoltar	**74.56**	DStar	63.96
Op2	**74.56**	Tarantula	61.00
Ochiai	**74.44**	Barinel	14.28
Mountford	**72.44**	Ar. Mean	13.92
Br.-Banq	**71.92**	Phi	13.92
Russel Rao	**70.92**	Cohen	13.80
B.-U & B	69.80	Ochiai2	11.76
S. Match	65.44	Pierce	6.28

**Table 9:** Average values for EXAM scores in average-case scenarios for each case study (MRC: multi-rule contracts; SRC: single-rule contracts)

RSS2ATOM - SRC		UML2ER - MRC		UML2ER - SRC	
Technique	Val	Technique	Val	Technique	Val
Pierce	0.439	Kulcynski2	**0**.**270**	Kulcynski2	**0**.**237**
Kulcynski2	0.617	Zoltar	**0**.**270**	Zoltar	**0**.**237**
Zoltar	0.617	Ochiai	**0**.**270**	Ochiai	**0**.**237**
Ochiai	0.617	Op2	**0**.**270**	Op2	**0**.**237**
Br.-Banq	0.623	Br.-Banq	**0**.**271**	Russel Rao	**0**.**240**
B.-U & B	0.623	B.-U & B	**0**.**275**	Br.-Banq	**0**.**245**
Russel Rao	0.625	Russel Rao	**0**.**275**	B.-U & B	**0**.**248**
Ochiai2	0.642	S. Match	**0**.**280**	S. Match	**0**.**253**
Op2	0.643	Rog & Tan	**0**.**280**	Rog & Tan	**0**.**253**
S. Match	0.660	Mountford	0.306	Mountford	**0**.**287**
Rog & Tan	0.660	Tarantula	0.434	Tarantula	0.484
Tarantula	0.665	Cohen	0.512	Cohen	0.512
DStar	0.678	Ar. Mean	0.512	Ar. Mean	0.512
Mountford	0.679	Phi	0.512	Phi	0.512
Cohen	0.759	Ochiai2	0.517	Ochiai2	0.538
Ar. Mean	0.759	DStar	0.554	Barinel	0.557
Phi	0.759	Barinel	0.590	DStar	0.696
Barinel	0.802	Pierce	0.758	Pierce	0.835

GM2Auto - MRC		GM2Auto - SRC		UML2Kil - MRC	
Technique	Val	Technique	Val	Technique	Val
Br.-Banq	0.458	Kulcynski2	**0**.**266**	Kulcynski2	**0**.**210**
S. Match	0.458	Zoltar	**0**.**266**	Zoltar	**0**.**210**
Kulcynski2	0.458	Ochiai	**0**.**266**	Ochiai	**0**.**210**
Zoltar	0.458	Russel Rao	**0**.**266**	Op2	**0**.**210**
Ochiai	0.458	Op2	**0**.**266**	Mountford	**0**.**211**
Op2	0.458	Br.-Banq	**0**.**271**	Russel Rao	**0**.**234**
Russel Rao	0.458	B.-U & B	**0**.**271**	Br.-Banq	**0**.**272**
B.-U & B	0.458	S. Match	**0**.**271**	Tarantula	0.319
Rog & Tan	0.458	Rog & Tan	**0**.**271**	Cohen	0.338
Cohen	0.556	Mountford	**0**.**272**	Ar. Mean	0.338
Barinel	0.556	DStar	**0**.**274**	Phi	0.338
Ar. Mean	0.556	Tarantula	**0**.**276**	DStar	0.371
Phi	0.556	Ochiai2	0.553	B.-U & B	0.374
Ochiai2	0.556	Cohen	0.554	Barinel	0.376
Tarantula	0.556	Ar. Mean	0.554	Ochiai2	0.486
Mountford	0.611	Phi	0.554	S. Match	0.513
DStar	0.615	Pierce	0.569	Rog & Tan	0.513
Pierce	0.653	Barinel	0.832	Pierce	0.797

UML2Kil - SRC		F2P - MRC		F2P - SRC	
Technique	Val	Technique	Val	Technique	Val
Zoltar	**0**.**133**	Kulcynski2	**0**.**266**	Kulcynski2	**0**.**167**
Op2	**0**.**133**	Zoltar	**0**.**266**	Zoltar	**0**.**167**
Kulcynski2	**0**.**133**	Ochiai	**0**.**267**	Ochiai	**0**.**168**
Ochiai	**0**.**136**	Russel Rao	**0**.**269**	Mountford	**0**.**171**
Mountford	**0**.**138**	Mountford	**0**.**279**	Russel Rao	**0**.**174**
Br.-Banq	**0**.**155**	Op2	**0**.**286**	Op2	**0**.**175**
Russel Rao	**0**.**168**	Br.-Banq	0.316	Br.-Banq	**0**.**203**
B.-U & B	**0**.**209**	DStar	0.418	B.-U & B	**0**.**246**
Tarantula	0.317	Tarantula	0.419	S. Match	**0**.**298**
S. Match	0.321	B.-U & B	0.436	Rog & Tan	**0**.**298**
Rog & Tan	0.321	Barinel	0.448	Tarantula	0.430
Ar. Mean	0.324	Cohen	0.454	Cohen	0.449
Phi	0.324	Ar. Mean	0.454	Ar. Mean	0.449
Cohen	0.325	Phi	0.454	Phi	0.449
Ochiai2	0.374	S. Match	0.518	Barinel	0.452
Barinel	0.374	Rog & Tan	0.518	Ochiai2	0.502
DStar	0.467	Ochiai2	0.620	DStar	0.602
Pierce	0.862	Pierce	0.752	Pierce	0.858

Bold values indicate the scores are acceptable results for an EXAM score

#### Evaluation metrics for answering RQ3

For answering this RQ about the speed of the approach in Sect. 5.3, we require execution time measurements. We collect the number of seconds needed for each transformation to have the contracts verified and for the results to be processed to produce EXAM scores for all techniques. These timings are presented in Table 11, taken to be the average of three execution runs.

### Prototypical implementation

Our *SBFL-Verif* approach is supported by a prototypical implementation. This implementation has two components: a) the SyVOLT verification tool [64], and b) the EXAM score calculator [63].

As mentioned in Sect. 2.3, SyVOLT is a contract verification tool which builds the symbolic execution space of a transformation and then proves contracts on that space. As output, SyVOLT produces a set of *path conditions* (detailing which rules have symbolically executed), and whether each contract is *satisfied*, *not satisfied*, or *not applicable*. For this work, SyVOLT has also been extended to perform mutation on model transformations (cf. Section 4.2.3). An XML file containing the results of contract satisfaction on each path condition can then be easily produced for each mutant automatically.

The second component in our implementation is a Java-based program to read in the XML files produced from SyVOLT, and compute the EXAM scores for each suspiciousness technique. Specifically, for each mutant the *coverage matrix* is generated of each path condition and contract results, the *suspiciousness technique equations* are evaluated, the *ranking of the suspicious rules* is produced, and the *EXAM score* is calculated based on the known faulty rule.

As this implementation has mostly been developed to support this paper, the calculator tool produces comma-separated value (CSV) files which summarize the suspiciousness techniques in the best-, worst- and average-case scenarios. This would not be useful in the methodology suggested in Sect. 3.3. However, when the calculator is used as in the methodology, an easy-to-interpret CSV containing the suspiciousness ranking of the rules would be produced.

#### Execution environment

All the experiments have been executed on a desktop PC running 64-bit Ubuntu 19.10, with an Intel i5-4570 3.6 GHz quad-core processor, and 16 GB of RAM. The software used was Eclipse Modeling Tools version 2019-12, and Python 3.4.3.

## Evaluation results and critical discussion

This section answers the research questions posed in Sect. 4.1 and provides a critical discussion of the results. We also investigate the threats to validity for our experiments.

### RQ1

Question: *Is* SBFL-Verif *powerful enough to discover faulty rules effectively?*

As mentioned in Sect. 4.3.1, this research question is answered by examining the EXAM score for the suspiciousness values produced by the different suspiciousness techniques for all model transformations. Specifically, the data in Table 7 has been obtained after the automatic construction of 2499 suspiciousness tables with our approach. This means that, out of the total of 91 contracts defined (cf. Table 5) and the 4852 mutants produced (cf. Table 6), there are 2499 mutants in which at least one contract is satisfied for at least one path condition and at least one contract fails for at least one path condition. That is, there are 2499 scenarios in which our approach has been applied and evaluated for locating the faulty rule.

In Table 7, we have highlighted with bold font those EXAM scores whose mean is below 0.3 in the average-case scenario. Results have been grouped by increasing value of this measure. We can see that there are eight techniques for which the mean of the EXAM score in the average-case scenario is below 0.3. These are *Zoltar*, *Kulcynski2*, *Ochiai*, *Op2*, *Russel Rao*, *Mountford*, *Braun-Banquet* and *Baroni-Urbani & Buser*.

The differences of the average mean values in the best- and worst-case scenarios do not differ much among these techniques, and the values for the standard deviation of these eight techniques are also quite similar. For this reason, having only the data in this table, it is not possible to state with certainty which of these techniques is better. However, for now we can state that these *eight techniques behave effectively* in the *SBFL-Verif* approach which uses fault localization in the context of model transformation verification.

The percentage of cases in which the faulty rule is located within the top-three ranked rules is displayed in Table 8. Techniques are ordered by a decreasing value of the percentage, and values above 70% have been highlighted. We can see that the order of the techniques is similar to the one obtained in Table 7. Since the value for technique *Baroni-Urbani & Buser* is below 70%, we remove this technique from the previous list of eight techniques. Furthermore, *Kulcynski2*, *Zoltar*, *Op2* and *Ochiai* seem to provide the best results, since they are placed in the top four both in Table 7 and Table 8.

**Table 10:** Average values for EXAM scores in average-case scenarios for each mutation

Add Assoc.		Add Back. Link		Add element		Delete element	
Technique	Val	Technique	Val	Technique	Val	Technique	Val
Kulcynski2	**0**.**206**	Russel Rao	**0**.**276**	Kulcynski2	0.315	Zoltar	**0**.**17**
Mountford	**0**.**206**	Op2	**0**.**289**	Zoltar	0.315	Op2	**0**.**17**
Zoltar	**0**.**206**	Br.-Banq	0.342	Ochiai	0.315	Kulcynski2	**0**.**17**
Ochiai	**0**.**206**	S. Match	0.342	Mountford	0.317	Ochiai	**0**.**17**
Op2	**0**.**206**	Kulcynski2	0.342	Russel Rao	0.33	Russel Rao	**0**.**186**
DStar	**0**.**206**	Mountford	0.342	Op2	0.347	Mountford	**0**.**187**
Russel Rao	**0**.**26**	Zoltar	0.342	Br.-Banq	0.359	Br.-Banq	**0**.**198**
Cohen	**0**.**267**	Ochiai	0.342	Tarantula	0.369	B.-U	**0**.**247**
Ar. Mean	**0**.**267**	B.-U	0.342	DStar	0.392	S. Match	0.305
Phi	**0**.**267**	Rog & Tan	0.342	B.-U	0.409	Rog & Tan	0.305
Br.-Banq	**0**.**269**	DStar	0.342	Ar. Mean	0.429	Tarantula	0.393
Tarantula	**0**.**273**	Cohen	0.553	Phi	0.429	Cohen	0.414
Barinel	0.313	Barinel	0.553	Cohen	0.43	Ar. Mean	0.414
Ochiai2	0.398	Ar. Mean	0.553	Barinel	0.454	Phi	0.414
B.-U	0.403	Phi	0.553	Ochiai2	0.47	Barinel	0.449
S. Match	0.552	Tarantula	0.553	S. Match	0.563	Ochiai2	0.487
Rog & Tan	0.552	Ochiai2	0.73	Rog & Tan	0.563	DStar	0.541
Pierce	0.777	Pierce	0.776	Pierce	0.654	Pierce	0.853

Delete equation		Modify equation		Retype Assoc.		Retype element	
Technique	Val	Technique	Val	Technique	Val	Technique	Val
Kulcynski2	**0**.**197**	Russel Rao	**0**.**133**	Kulcynski2	**0**.**162**	Kulcynski2	**0**.**19**
Zoltar	**0**.**197**	Kulcynski2	**0**.**138**	Zoltar	**0**.**162**	Zoltar	**0**.**19**
Op2	**0**.**197**	Zoltar	**0**.**138**	Op2	**0**.**162**	Op2	**0**.**19**
Ochiai	**0**.**198**	Op2	**0**.**138**	Ochiai	**0**.**163**	Ochiai	**0**.**192**
Russel Rao	**0**.**208**	Mountford	**0**.**141**	Mountford	**0**.**169**	Mountford	**0**.**2**
Br.-Banq	**0**.**209**	Ochiai	**0**.**141**	Br.-Banq	**0**.**184**	Russel Rao	**0**.**207**
Mountford	**0**.**217**	Br.-Banq	**0**.**191**	Russel Rao	**0**.**196**	Br.-Banq	**0**.**235**
B.-U	**0**.**246**	Tarantula	0.351	B.-U	**0**.**233**	B.-U	**0**.**299**
S. Match	0.305	B.-U	0.356	S. Match	0.309	S. Match	0.369
Rog & Tan	0.305	Cohen	0.375	Rog & Tan	0.309	Rog & Tan	0.369
Tarantula	0.429	Ar. Mean	0.375	Tarantula	0.333	Tarantula	0.402
Cohen	0.446	Phi	0.375	Cohen	0.347	Cohen	0.413
Ar. Mean	0.446	Barinel	0.413	Ar. Mean	0.347	Ar. Mean	0.413
Phi	0.446	DStar	0.432	Phi	0.347	Phi	0.413
Ochiai2	0.48	S. Match	0.475	Barinel	0.383	Barinel	0.436
Barinel	0.482	Rog & Tan	0.475	Ochiai2	0.404	DStar	0.475
DStar	0.612	Ochiai2	0.575	DStar	0.501	Ochiai2	0.512
Pierce	0.843	Pierce	0.89	Pierce	0.842	Pierce	0.841

Bold values indicate the scores are acceptable results for an EXAM score


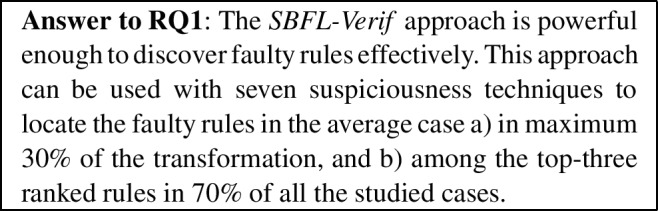

**Table 11:** Number of mutants for each case study, time taken for experimentation and scoring, and a per-mutant estimate

Case study	Num. Muts.	Contr. type	Verif. time (s)	Score time (s)	Time Per Mut.(s)
*RSS-to-*	232	MRC	-	-	-
*ATOM*		SRC	296	1	1.28
*UML-*	191	MRC	261	2	1.38
*to-ER*		SRC	262	3	1.39
*GM-to-*	840	MRC	1189	8	1.43
*AUTOSAR*		SRC	864	5	1.03
*UML-to-*	2658	MRC	9896	128	3.77
*Kiltera*		SRC	11,261	122	4.28
*Families-*	931	MRC	2025	29	2.21
*to-Persons*		SRC	2022	26	2.20

### RQ2

Question: *How do the different inputs to* SBFL-Verif (*model transformation*, *contract type*, *and specific* *mutations*) *affect the results?*

This research question is broken down into three sub-questions.

#### RQ2.1

Question: *How does the model transformation under test affect the results?*

As the model transformations examined in this work are of varying sizes and complexity, we here present the results of the *SBFL-Verif* approach for each transformation separately. The EXAM scores are presented in Table 9, where the columns are for each transformation and contract type and the rows are each suspiciousness calculation technique. Figure 7 then presents this information as boxplots which provide five-number summaries of the distribution of values. For both Table 9 and Fig. 7, lower EXAM scores are better.

Through examination of these results, we have divided the results into two groups. The first group is two transformations where the *SBFL-Verif* approach has been unsuccessful, and the other group where it has been successful.

*Negative Cases* The fault localization results for the *RSS-to-ATOM* and *GM-to-AUTOSAR* transformations are very poor. In the case of the single-rule contracts (SRC) for both transformations, the entire transformation may have to be searched for finding the faulty rule. This can be seen in Fig. 7 by the boxes and dots which extend to an EXAM score near 1.0. For the *GM-to-AUTOSAR* transformation results with the multi-rule contracts (MRC), the techniques consistently identify that half the rules must be searched, which is seen with the boxplots grouped near an EXAM score of 0.5. This outcome is also undesirable as the user will have to search many rules.

We conclude that these results are not useful, and therefore the *SBFL-Verif* approach is not applicable to these transformations. However, it is important to determine why the technique does not apply. The answer most likely lies within the symbolic execution technique used to prove the contracts.

Recall that the SyVOLT prover generates *path conditions* which represent how rules can combine in a transformation (Sect. 2.3). These path conditions are then matched by the contracts to see in which conditions the contracts are satisfied, and when they are not satisfied. Our hypothesis for these poor results is that if there is an insufficient number of path conditions, then there is not enough information for the *SBFL-Verif* approach to identify the faulty rule. That is, there will be a lack of *satisfaction cases* (contracts $\times$ path conditions) needed to generate a robust coverage matrix for SBFL (Sect. 2.4). We presume that any SBFL approach would struggle to deal with this lack of coverage and that having more path conditions, and thus more satisfaction cases for the coverage matrix, would improve the efficacy of our approach [80]. Future work will attempt to precisely identify the relationship between the number of path conditions and the EXAM score quality.

To gain an indication of how many path conditions could be sufficient, Table 3 presents the number of path conditions generated for each transformation. The *RSS-to-ATOM* and *GM-to-AUTOSAR* transformations produce only five path conditions each, while the others produce over twenty. The path conditions produced are a consequence of how the transformation rules combine and overlap, which is extremely difficult to predict (see Sect. 2.3). Future work could be to adjust the underlying technique to generate more path conditions by modifying the abstraction relation used to map path conditions to transformation executions [48].

*Positive Cases* For the remaining transformations (*UML-to-ER*, *UML-to-Kiltera* and *Fam-to-Per-Ex*), there are several techniques with EXAM score values below 0.3. For these techniques, the successful results are shown graphically in Fig. 7 boxplots, where the lines and rectangles are of a reasonable length and relatively tightly grouped near the bottom. This suggests that the EXAM score values obtained are relatively similar no matter the contract and mutant used.

In particular, we highlight the excellent results for the single-rule contracts (SRC) for the *UML-to-Kiltera* and *Fam-to-Per-Ex* transformations. In these cases, multiple techniques provide information such that the faulty rule could be found by searching in only 14 or 17 percent of the transformation (three rules and four rules, respectively).

From these results, we conclude that our SBFL approach is applicable to these three transformations, ranking the faulty rule within at most one-third of the transformation. The top-three techniques seem to be *Kulcynski2*, *Zoltar* and *Ochiai*, closely followed by *Op2*.
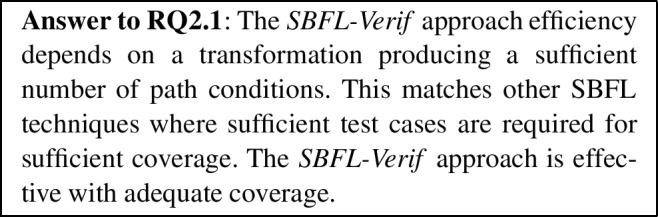


#### RQ2.2

Question: *How do the contracts affect the results quality?*

Section 4.2.2 describes how we consider two types of contracts: a) *multi-rule contracts* (MRC), which involve elements from multiple rules in the transformation, and b) *single-rule contracts* (SRC), which are mirrored versions of the rules. From the results in Table 9 and Fig. 7, the SRCs provide a slightly better EXAM score on almost all techniques compared to the MRCs. Building these SRCs also allowed us to test our approach on the *RSS-to-ATOM* transformation which did not have usable MRCs.
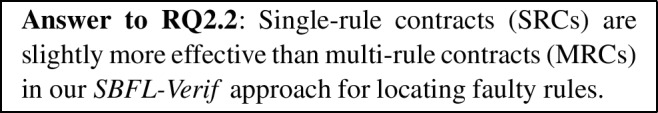


#### RQ2.3

Question: *How do specific faults affect the results quality?*

In this work, we have defined eight mutation operators on DSLTrans transformations, which in total have produced 4852 mutants (cf. Section 4.2.3). An immediate question is then how well the EXAM techniques are able to detect each type of mutation. Table 10 presents the EXAM scores in the average-case scenario for the transformations where the SBFL approach was effective (the *UML-to-ER*, *UML-to-Kiltera*, and *Fam-to-Per-Ex* transformations, as explained in Sect. 5.2.1). We note that the top SBFL techniques are in most cases quite effective, producing scores at or below 0.20. It is only for the mutations of adding a backward link and adding elements where the techniques perform poorly.

The reasons why these specific mutations provide poor results is a topic of further investigation, but an initial examination suggests that these mutations are unlikely to falsify both the MRCs and SRCs used in the current evaluation.^8^ The current set of contracts used could be considered incomplete as it cannot sufficiently detect these mutations. Thus, the *SBFL-Verif* approach (or presumably any fault localization approach) is unable to detect these errors and locate the rule in question. Future work will attempt to resolve this issue, either by producing contracts which are falsified by these mutants or by adjusting the *SBFL-Verif* approach to better localize these faults.
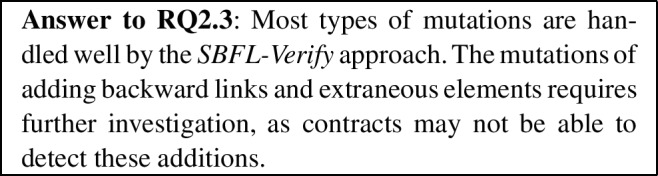


### RQ3

Question: *Is the* SBFL-Verif *approach fast enough to discover faulty rules efficiently?*

The focus of the current paper is on the development of and initial results for the *SBFL-Verify* approach. As such, our focus was not on optimizing execution times or other resource consumption. However, it is important to have some measure of the efficiency of the current implementation. Table 11 details the number of seconds needed for each transformation^9^ to have the contracts verified and for the results to be processed to produce EXAM scores for all techniques.

The times listed may seem inefficient, as the lowest time taken is 263 s and the highest time taken is 11,383 s (over three hours.) However, this is the total time taken for verification and scoring for all mutants, which number in the thousands. In the methodology we propose in Sect. 3.3, the developer would be examining one transformation, corresponding to one mutant. Therefore, a rough estimate is provided in the right-most column of Table 11, where the combined time per mutant in our transformations ranges from $869/840 = 1.03$ seconds for the fastest case (*GM-to-AUTOSAR*) to $11383/2658 = 4.28$ seconds for the slowest case (*UML-to-Kiltera*).

We thus consider this time taken to allow for a reasonably efficient process to be applied in debugging scenarios with the human-in-the-loop. That is, the delay is short enough for the user to pause for the results and continue developing the transformation without losing attention.
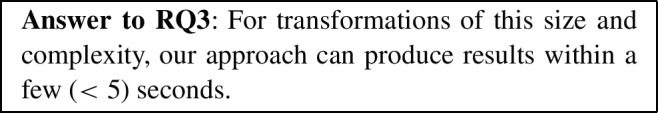


## Discussion and threats to validity

After performing this thorough evaluation, this section is devoted to discuss some interesting findings. As a main result, we can conclude that *combining symbolic execution-based contract checking on model transformations with spectrum-based fault localization in the* SBFL-Verif *approach can provide effective localization of faulty rules*.

The caveat to this is that the symbolic execution must produce a sufficient number of path conditions such that the SBFL techniques have sufficient coverage. However, the good news is that (a) more path conditions are usually produced as the size of a model transformation grows, and (b) the path conditions can be computed very fast. A pre-check in our methodology can thus check whether a certain number of path conditions is produced or not. In the latter case, a warning could be shown to the model transformation engineer that the *SBFL-Verif* approach may not lead to effective tracking of the faulty rules.

**Table 12:** Average values for EXAM score in AC scenario excluding *GM-to-AUTOSAR* and *RSS-to-ATOM* case studies

Technique	Val	Technique	Val
Zoltar	**0**.**214**	Rog & Tan	0.364
Kulcynski2	**0**.**214**	Tarantula	0.401
Ochiai	**0**.**215**	Ar. Mean	0.432
Op2	**0**.**219**	Phi	0.432
Russel Rao	**0**.**226**	Cohen	0.432
Mountford	**0**.**232**	Barinel	0.466
Br.-Banq	**0**.**244**	Ochiai2	0.506
B.-U & B	**0**.**298**	DStar	0.518
S. Match	0.364	Pierce	0.810

Bold values indicate the scores are acceptable results for an EXAM score

If there are a sufficient number of path conditions, the *SBFL-Verif* approach is quite effective. For example, in Table 12, we present the EXAM score averages in the average-case scenario *without considering the* *GM-to-AUTOSAR* *and* *RSS-to-ATOM* *transformations*. Comparing Table 12 against Table 9, we see that the EXAM scores improve even further. In particular, there are four SBFL techniques (*Zoltar*, *Kulcynski2*, *Ochiai* and *Op2*) where the EXAM score is below 0.22, which we consider to be a good fault localization score. The remaining eleven techniques are not recommended to be used in the context of fault localization in model transformation verification.

### Comparison of SBFL-verif and SBFL-test

In those cases where the application of *SBFL-Verif* does not provide effective-enough results, an alternative can be the application of spectrum-based analysis following a dynamic approach [69] (*SBFL-Test*, cf. Section 2.4). The challenge with the *SBFL-Test* approach is that a large number of test cases should be provided to have useful coverage for the suspiciousness techniques. For example, the work in [69] reports results using 100 test cases as input for each model transformation.

In any case, our *SBFL-Verif* approach and the *SBFL-Test* approach [69] can be complementary. If contracts are available, and the symbolic execution process produces a sufficient number of path conditions, then no test cases need to be created and it is straightforward to apply our approach. On the other hand, if a large number of test cases are generated (a challenging problem [10, 11]), then the *SBFL-Test* approach [69] can be applied. An interesting line of future work is the generation of test cases from satisfaction cases: when our approach does not provide accurate-enough results with the available satisfaction cases, input models could be obtained from path conditions, achieving an interplay between both approaches.

To briefly compare the efficiency of the two approaches, the conclusions found in Sect. 5 for *SBFL-Verif* and those from [69] for *SBFL-Test* are similar, although a thorough evaluation of the scores based on the model transformations, contracts and mutants is not reported in that work. We also note that the *SBFL-Test* approach is reported to take around 4 and 75 s per mutant [69], while our *SBFL-Verif* approach takes around 1 to 5 s per mutant.

In the *SBFL-Test* approach, the best techniques allow the user to inspect a maximum of three rules to locate the bug in around 74% of the cases. This conclusion is very similar to the one we reported in Sect. 5.1, where we conclude that the best techniques are able to locate the faulty rule in the top-three over 70% of the time for all case studies analyzed.

EXAM scores reported in [69] are slightly lower than the ones reported in our study. In particular, the top two techniques in that work (*Mountford* and *Kulcynski2*) provide average EXAM scores of 0.205 and 0.207. However, these results are not consistent across transformations, as when comparing the *UML-to-ER* transformation studied in both works. The *SBFL-Verif* approach leads to EXAM scores below 0.3 for this transformation (see Table 7), while the *SBFL-Test* approach leads to values above 0.3 as reported in Table 7 of [69].

Interestingly, *Kulcynski2*, *Ochiai*, *Zoltar* and *Mountford* are the most efficient techniques for *SBFL-Test* [69]; while *Kulcyinski2*, *Ochiai*, *Zoltar* and *Op2* are the most efficient techniques for *SBFL-Verif* as reported by our evaluation. We see that the only difference is the switch of *Mountford* and *Op2*; however, these techniques have relatively good results in both approaches. Thus, this work confirms the previously reported results in [69], although different model transformation languages, contract languages and model transformations are used.

### Threats to validity

Wohlin et al. [77] identified four types of threats which have to be discussed in the context of our study.

#### Conclusion validity threats

The first threat is drawing incorrect conclusions from the experimental data. To mitigate this, we have applied statistical analysis by comparing the mean and standard deviation of all case studies, and have also considered the differences in the EXAM scores of best- and worst-case scenarios. Box-plot charts for all case studies have been obtained and analyzed too. Furthermore, we have applied a deep analysis by studying how the model transformations under test, the contracts and the mutants used in the experiments influence the results. Finally, our prototypical implementation and the evaluation data are available online for replication of our study in the companion materials of this paper [63, 64].

#### Construct validity threats

A single metric, namely the EXAM score, has been selected to evaluate the effectiveness of the SBFL techniques and the impact of the transformation, mutation and contract types on the results. In addition to the EXAM score, alternative metrics have been proposed [80], e.g., the T-score [38], P-score [83] and N-score [28] to mention just a few. We selected the EXAM score because (*i*) it is a well-accepted metric, (*ii*) is often used in previous work [80], and (*iii*) is straightforward to compute and easy to understand.

#### Internal validity threats

We are now discussing threats that might affect the results of our study. As the first threat, we discuss the usage of a fixed set of 91 contracts (cf. Table 5). This threat is minimized by the construction of contracts which provide a good coverage of the transformation implementations and are of two different types, namely multi- and single-rule contracts (cf. Sect. 4.2.2). Indeed, the single-rule contracts are built from the transformation rules, assuring that they cover the complete model transformation.

Another threat to internal validity is the set of model transformation mutation operators used in the evaluation. Our mutation operators have been created automatically and are based on the DSLTrans meta-model and the authors’ experience with DSLTrans transformations faults [59], so that they cover a wide variety of aspects of the language. A total amount of 4852 mutants have been generated (cf. Table 6). From these mutants, the 91 contracts defined cause 2499 of them to fail, which means that 2499 mutants have been used for computing the suspiciousness-based rankings used in the evaluation. We consider this is a large-enough set of mutants to believe that the results obtained are not affected by the specific mutants used, so this threat is sufficiently mitigated. Another threat in our mutation strategy is that only one rule at a time is mutated. The presence of multiple mutations in a rule may require further modification to the suspicousness techniques [21].

#### External validity threats

This section mentions threats that might negatively affect the generalization of our results. As a first threat, we have to point to the fixed set of five case studies which are used in our study. To mitigate this threat, we selected model transformations having different size and complexity matching those in the ATL zoo, coming from different domains and employing different transformation language features in their implementations (cf. Table 3). Furthermore, the transformations used are larger than a majority of relevant ATL zoo transformations [46], with the *UML-to-Kiltera* and *Fam-to-Per-Ex* transformations larger than 70% of this selection of the zoo. While larger transformations exist, we consider these to be a threat to the *scalability* of the approach rather than the *effectiveness*. Finally, we have also used a case study from industry, namely the *GM-to-AUTOSAR* case study.

Second, we employed 18 techniques for suspiciousness-based ranking of model transformation rules. However, there might be better techniques, i.e., providing higher EXAM scores, which we did not consider in our study. To counteract this threat, we have used the same techniques as proposed in previous work [69].

Third, we have realized our approach for the DSLTrans language and SyVOLT contract prover, since they provide a powerful toolkit for transformation verification using symbolic execution. However, as discussed in Sect. 3.3.2, our approach may produce similar results for other model transformation languages and verification tools which provide the same analysis capabilities and outputs, i.e., path conditions. Any such tool may serve as input for our SBFL computation (cf. Steps 2 and 3 in Fig. 5), which constructs the coverage matrices and error vectors, and computes the suspiciousness-based rankings.

Finally, we have to mention that our approach locates bugs at rule level only, such as related approaches do. This means, it would be more challenging for the transformation engineer to locate a bug in a large rule than in a small rule. While the debugging output provided by SyVOLT attempts to assist in further localization by presenting the elements present in the contract which are missing in the path condition, this is only a first attempt [51]. To mitigate this threat, it would be interesting to study as future work the possibility to locate bugs in specific elements in the rule.

## Related work

There is significant previous work in the general field of model transformation validation, verification and debugging. For instance, consider [29, 36, 39, 58, 74] for concrete approaches and [4, 14, 21, 24, 55] for surveys. Our presented approach focuses on employing SBFL for symbolic execution techniques to track the faulty rules and, up to our knowledge, is the first work in this line. Therefore, with respect to the contribution of this paper, we discuss two threads of related work: (*i*) fault localization approaches which use static techniques, i.e., not requiring to execute test suites, and (*ii*) works that use dynamic approaches which require to execute test suites. Please note that the presented *SBFL-Verif* approach of this paper falls in the first category. Finally, a contribution of this work has also been the implementation of automatic mutation of input DSLTrans transformations within the SyVOLT verification tool (cf. Section 4.2.3). For this reason, we add a third categorization of works related to the definition of mutants in an MDE context.

### Static fault localization

The work of Burgueño et al. [13] compares the elements of transformation contracts to the rules in the transformation in a static way. In particular, this is done by computing the footprints of the contracts and rules in their associated source and target meta-models. The main idea is to present to the transformation engineer which rules are causing contracts to fail by matching the used elements and associations in the rules and contracts based on the computed meta-model footprints. The approach shows good performance when the footprints of the transformation rules are diverse, but in cases where the rule footprints are very similar, the fault tracking is more challenging as more rules have to be potentially investigated.

Cheng and Tisi [15, 17] present a static approach based on deductive reasoning for tracking guilty rules in ATL transformations. OCL conditions are decomposed into sub-goals using a deductive rule-based approach. Unverified sub-goals can then be traced to transformation rules, allowing for a static slicing approach to provide users with the specific potentially faulty rules. Their approach focuses on the decomposition of the verification contracts and tracing the sub-goal failures back to the faulty rules. In contrast, our symbolic execution technique focuses on generating the state space of the transformation as path conditions, and then using the contract satisfaction result to trace back to the faulty rules which were symbolically executed in that path condition.

Cuadrado et al. [19] presented a technique for static analysis of ATL transformations. In particular, they are using type inference in combination with other techniques to report potential syntactic problems in the transformation. In addition, OCL constraints are generated in order to again generate “witness” input models which should allow to locate the fault. Furthermore, in [20] also presents a method and system to propose quick fixes for transformation errors.

Similar to these mentioned approaches, no input models are necessary in our approach to perform fault localization, which eases its applicability. However, the main difference of our work to the aforementioned related ones is that we manage to locate faulty rules by combining symbolic execution and spectrum-based techniques. In fact, we not only perform a static reasoning based on the content of the model transformation, like previous works do, but we use the information on the path conditions taken from symbolic execution to aid in the effective detection of bugs.

There are multiple works that propose to convert model transformations to a formal domain where verification can be carried out automatically [4, 24, 55, 71]. However, these works tend to focus on the conversion to the formal domain, and not on the detection of bugs.

### Dynamic fault localization

Locating faulty transformation rules by using a dynamic approach, i.e., requiring an execution of the model transformation for particular test cases, has been subject to several investigations in recent years. Hibberd et al. [31] discussed so-called forensic debugging techniques for model transformations by resorting on the traces produced as a side product of model transformation executions. They employ the traces for reasoning about the relationship between source and target model elements as well as the transformation rules. They also proposed debugging questions formulated as model queries which are executed on the traces (as they are also represented as models, so-called trace models). Following this line, in [75], OCL-based queries are employed for the backwards debugging of model transformations. A dedicated runtime model incorporating the trace models between source and target models is constructed which is the basis for querying the history of a transformation execution for locating faults. Another approach for locating faults in model transformations by exploiting trace models between source and target models is presented in Aranega et al. [5]. Finally, a dynamic slicing approach for model transformations is presented in [72]. This approach can be used to produce slices of model transformations which may be subsequently applied for fault localization purposes.

The work by Troya et al. [69] pioneered the application of SBFL to the domain of model-driven engineering, by applying SBFL techniques for locating faults in model transformations in the *SBFL-Test* approach. The *SBFL-Verif* approach we propose in this paper follows this line of research. However, in the previous work of Troya et al. [69], transformations need to be executed, for which a test suite must be developed and maintained. After the model transformation finishes, the coverage matrix is built by using the information about the model transformations available in the execution traces. In the current *SBFL-Verif* approach, however, we do not need to execute the transformation for locating faults. Instead, we apply symbolic execution for building all possible path conditions for constructing the coverage matrix. This *SBFL-Verif* approach does not execute the transformation directly, and is thus especially useful in the case where bugs in a transformation cause execution errors and the transformation cannot finish properly. Despite being independent approaches, *SBFL-Verif* and *SBFL-Test* can be complementary as discussed in Sect. 6.1.

Other approaches which uses test models for performing SBFL for model transformations are presented in [22, 37, 56]. Du et al. proposed to combine SBFL with metamorphic testing as previously proposed in [70]. This allows the application of SBFL without the need to count on an oracle, whereas our work relies on contracts as an oracle. Li et al. proposed to use weighted test models as well as weighted rule coverage to improve the performance of SBFL, but follow the classical approach of SBFL using large test suites [37]. Rodriguez-Echeverria et al. develop a novel approach based on precision and recall metrics calculated for every output pattern when executing test models [56]. Combined with traceability information, repair actions are then suggested on particular rules, allowing for effective fault localization and repair.

### Mutants in MDE contexts

There has been extensive work on the definition and automatic generation of mutants in order to apply mutation analysis in MDE contexts [67]. One research line is the mutation of OCL expressions which is of general interest since OCL is an important MDE standard. Jin and Lano [33] propose a set of mutation operators for the OCL standard library and classify them into different groups. Clarisó and Cabot [18] also deal with the mutation of OCL expressions by mutating integrity constraints expressed in OCL in a structured way. However, there is still a need for the integration of the automatic generation of OCL mutants in MDE tools.

Another research line is the generation of mutants for a given model conforming to a meta-model. Sen and Baudry [62] presented an approach to derive primitive mutation operators from meta-models by interpreting them as graph-grammar rules [23]. These rules are automatically generated by a model transformation which considers the addition of model elements, relationships and attributes. Gómez-Abajo et al. [26, 27] presented WODEL, a dedicated language for the specification and automatic generation of model mutants. WODEL considers the following operators: deletion and addition of elements, element selection strategies and mutation compositions.

The research line most related to the mutants we have defined and automated for DSLTrans is of course the definition of mutants for other model transformation approaches. Mottu et al. [44] pioneered the work on mutation analysis for model transformations. They define generic mutation operators for model transformations reflecting basic operations such as model navigation, filtering, output model creation and input model modification. Aranega et al. [6] turned the mutation operators proposed by Mottu et al. [44] into mutations for the Kermeta language. The presented mutation operators by Kahn and Hassine [35], Troya et al. [65], Sánchez-Cuadrado et al. [57] and Guerra et al. [30] are all defined for ATL model transformations. In particular, the work by Guerra et al. [30] revises the mutation operators proposed in the literature by the different authors and collect them in a consolidated catalogue of mutation operators for ATL.

While there have been several approaches to automate mutation analysis for different contexts as has been discussed in the previous paragraphs, we are not aware of any existing approach for DSLTrans. However, we followed general strategies, e.g., as outlined by Mottu et al. [44], and interpreted them in the context of DSLTrans, a graph pattern based transformation approach.

## Conclusion

In this paper, we have introduced the first (to the best of our knowledge) approach to combine a) a symbolic execution contract-based transformation verification approach with b) spectrum-based fault localization (SBFL) such that faulty rules can be suggested to the user. This *SBFL-Verif* approach allows a user to perform fault localization without relying on test cases, thus avoiding the burden of creating and maintaining test suites of sufficient size, quality and coverage.

The implementation of the *SBFL-Verif* approach described here utilizes the contract checker tool SyVOLT [64] to produce path conditions (which represent rule executions) and indicate which contracts are satisfied or not by each path condition. This output is fed into a number of suspiciousness-based ranking techniques, which rank the rules involved in the failure path conditions.

We have found that there are four very effective techniques for localizing faults, namely *Kulcyinski2*, *Zoltar*, *Ochiai* and *Op2*. The rankings they provide lead the user to inspect less than a quarter of the model transformation to locate the faulty rule in many case studies. As well, these and three other techniques (*Russel Rao*, *Mountford* and *Braun-Banquet*) are able to locate the faulty rule in the top-three over 70% of the time for all case studies analyzed.

These conclusions have been derived from studying how the model transformation under test, contracts and the model transformation faults (by creating mutants) affect the effectiveness of the SBFL techniques. Regarding the types of contracts, better results are generally obtained with single-rule contracts created from individual rules in the transformation, but multi-rule contracts also work well. Furthermore, our approach works well with most of the mutation types used in our study.

However, the approach also has some limitations when it comes to the number of path conditions which are produced for a transformation. For the coverage required for fault localization techniques, we estimate that at least one or two dozen path conditions is required for our approach to work effective. When this is not possible, we recommend applying the dynamic *SBFL-Test* approach for which the generation of test cases are necessary [69]. As future work, we aim to identify further the relationship between the number of path conditions and the quality of the EXAM scores, and the possibility of generating further path conditions on demand. In addition, we plan to investigate the use of path conditions to generate input models, allowing the interplay between both the *SBFL-Verif* and *SBFL-Test* approaches.

There are further lines of work that we would like to explore next. As single-rule contracts (SRCs) are mirrors of the rules, we plan to automate their creation. This may enable some degree of SBFL on transformations which do not yet have contracts. However, as mentioned in Sect. 4.2.2, not all SRCs are satisfied even on a correct version of the transformation. We leave as an open question how to combine (*i*) automatic SRC construction, and (*b*) managing the unknown contract verification status, during the construction process of the transformation. Finally, we will study the scalability of our approach and consider improvements such as incremental verification [16].

## Verifiability

Our prototype and all artifacts of the experiments are available online. First, the SyVOLT tool is available on [64], from which XML files with the results of the symbolic execution for all case studies, all contracts and all mutants have been obtained. Second, the outputs provided by SyVOLT are used as input for the spectrum-based fault localization implementation, provided in [63]. Instructions are provided for its execution. As well, the CSV files with the results generated for all case studies, contracts and mutants are provided.

## Footnotes

1 The set of rules involved is stored for each PC during the symbolic execution process.

2 These transformations and contracts can be built using visual editors previously developed [40, 48].

3 SyVOLT produces XML files containing each PC, the rules in each PC, and the contracts which fail on each PC.

4 AUTomotive Open System ARchitecture, AUTOSAR.org.

5 Dividing these numbers by the number of rules in the transformation provides a rough estimate of the complexity of the transformation. For example, the *Fam-to-Per-Ex* transformation has 46/19 = 2.4 input elements on average. This information is omitted from Table 3 for visual clarity.

6 For the *RSS-to-ATOM* transformation, the multi-rule contract case was not evaluated. This was due to the fact that only two multi-rule contracts are defined in [48]. One contract intentionally does not hold on the correct transformation (a *negative* contract). The remaining contract is too simple to produce results with our approach, as it was either satisfied in all mutants or was not applicable.

7 Once in the repository, select *SpecBased_FaultLoc_SyVOLT* and *Suspiciousness_Techniques.md.*.

8 The addition of a backward link may make explicit an implicit dependency already present in the rule. The addition of an extraneous element is often simply not tested for in contracts.

9 For the multi-rule contract results for the *RSS-to-ATOM* transformation, see footnote 6.
